# Comprehensive Profiling of Inflammatory Factors Revealed That Growth Differentiation Factor-15 Is an Indicator of Disease Severity in COVID-19 Patients

**DOI:** 10.3389/fimmu.2021.662465

**Published:** 2021-07-15

**Authors:** Xiangyun Teng, Jiaqi Zhang, Yaling Shi, Yuntao Liu, Yanqing Yang, Jinyong He, Shuhong Luo, Yile Huang, Yanxia Liu, Dongdong Liu, Yizhe Li, Shuangzhe Zhang, Ruo-Pan Huang, Dawei Wang, Jianhua Xu

**Affiliations:** ^1^ Department of Laboratory Medicine, Shunde Hospital of Guangzhou University of Chinese Medicine, Foshan, China; ^2^ Department of Laboratory Medicine, The Second Affiliated Hospital of Guangzhou University of Chinese Medicine, Guangzhou, China; ^3^ Department of Laboratory Medicine, Guangzhou Eighth People’s Hospital, Guangzhou, China; ^4^ Emergency Department, The Second Affiliated Hospital of Guangzhou University of Chinese Medicine, Guangzhou, China; ^5^ Research and Development Department, RayBiotech, Inc., Guangzhou, China; ^6^ Raybiotech Center, RayBiotech, Inc., Norcross, GA, United States; ^7^ Department of Pulmonary and Critical Care Medicine, Shunde Hospital of Guangzhou University of Chinese Medicine, Foshan, China

**Keywords:** COVID-19, inflammatory factors, GDF15, biomarker, disease severity

## Abstract

To systematically explore potential biomarkers which can predict disease severity in COVID-19 patients and prevent the occurrence or development of severe COVID-19, the levels of 440 factors were analyzed in patients categorized according to COVID-19 disease severity; including asymptomatic, mild, moderate, severe, convalescent and healthy control groups. Factor candidates were validated by ELISA and functional relevance was uncovered by bioinformatics analysis. To identify potential biomarkers of occurrence or development of COVID-19, patient sera from three different severity groups (moderate, severe, and critical) at three time points (admission, remission, and discharge) and the expression levels of candidate biomarkers were measured. Eleven differential factors associated with disease severity were pinpointed from 440 factors across 111 patients of differing disease severity. The dynamic changes of GDF15 reflect the progression of the disease, while the other differential factors include TRAIL R1, IGFBP-1, IGFBP-4, VCAM-1, sFRP-3, FABP2, Transferrin, GDF15, IL-1F7, IL-5Rα, and CD200. Elevation of white blood cell count, neutrophil count, neutrophil-lymphocyte ratio (NLR), Alanine aminotransferase and Aspartate aminotransferase, low lymphocyte and eosinophil counts in the severe group were associated with the severity of COVID-19. GDF15 levels were observed to be associated with the severity of COVID-19 and the dynamic change of GDF15 levels was closely associated with the COVID-19 disease progression. Therefore, GDF15 might serve as an indicator of disease severity in COVID-19 patients.

## Introduction

Coronavirus disease 2019 (COVID-19) resulting from severe acute respiratory syndrome coronary virus 2 (SARS-CoV-2) infection has been sweeping the globe at an unprecedented rate and is producing profound but still unfolding health and socio-economic impacts (1–4). Clinical experience to date has shown a high heterogeneity of COVID-19, with disease severity ranging from asymptomatic to mild to severe, resulting in a wide spectrum of clinical manifestations (cough, fever, myalgia, and malaise) that can eventually lead to acute respiratory distress syndrome (ARDS) or even cause death (5–7). However, it has been reported that many patients that became critically ill and died did not show severe clinical manifestations in the early stages of the disease, instead presenting with common symptoms such as mild fever, cough, or muscle soreness. These patients’ conditions suddenly deteriorated in the later stages of the disease or during recovery, where multiple organ failure, acute respiratory distress syndrome (ARDS), and death occurred within a very short period of time (2). Therefore, the research of biomarkers to monitor the COVID-19 process is particularly important.

Several studies have reported that cytokines are correlated with the progression of COVID-19. Cytokine synthesis, innate immune cell activation and adaptive immune response are key for rapidly targeting and combating the virus (8). However, uncontrolled excessive inflammation may lead to cytokine release syndrome (CRS), with impaired function causing tissue damage, immune cell apoptosis, and cytotoxicity (9). Cytokine storm is considered to be one of the major causes of ARDS and multiple-organ failure (10). Patients with severe COVID-19 have been reported to display unbalanced immune regulation (11, 12). Pro-inflammatory cytokines such as interleukin-1 (IL-1), IL-6, IL-8, and tumor necrosis factor alpha (TNF-α) have been found to be elevated in the sera of severely and critically ill COVID-19 patients, anti-inflammatory cytokines such as IL-10 have also been found in the sera of COVID-19 patients (13–15). Plasma IP-10, MCP-3 sTIM-3, HGF, IL-1α, IL-27, and PCT cytokines have also been reported to be related to the severity of the disease (16–19). The profile of cytokine gene expression by cDNA arrays and protein arrays are widely used in different infectious diseases to identify biomarkers (20–22).

To systematically explore potential severity-associated factors of COVID-19, we comprehensively analyzed the expression of 440 factors in asymptomatic, mild, moderate, severe, convalescent and healthy control groups (categorized according to COVID-19 disease severity). These factors encompass, cytokines, chemokines, growth factors, and secreted enzymes, etc., which relate to vascular growth, cell proliferation, cell migration, apoptosis, the inflammatory response as well as other processes. To further understand the dynamic changes of differentially expressed factors during the progression of COVID-19 in patients, sera were collected from patients at three time points (admission to the hospital, remission of symptoms, and discharge from the hospital) and the levels of the 440 factors were measured. To the best of our knowledge, this was the first study to exclusively characterize the factors associated with the severity and progression of COVID-19 using an antibody array profile. In order to provide a comprehensive dynamic characterization, longitudinal data from complete blood count, C-reaction protein (CRP), and liver function were also considered. We found eleven differentially expressed factors associated with disease severity, specifically TRAIL R1, IGFBP-1, IGFBP-4, VCAM-1, sFRP-3, FABP2, transferrin, GDF15, IL-1F7, IL-5Rα, and CD200. We further investigated GDF15 serum levels, and the results suggested that this cytokine may serve as an effective indicator of disease severity in COVID-19 patients.

## Materials and Methods

### Research Design and Patients

From 22 January 2020 to 13 May 2020, we carried out a retrospective study analyzing 111 patients diagnosed with COVID-19. These patients were diagnosed with real-time reverse transcriptase polymerase chain reaction (RT-PCR) oropharyngeal swab or nasopharyngeal swab tests upon admission to the Eighth People’s Hospital of Guangzhou. This hospital is the designated hospital for the treatment of COVID-19 in Guangzhou, and all COVID-19 patients in the current study were from this hospital.

To systematically explore potential severity associated factors, these 111 patients were categorized according to COVID-19 disease severity; including asymptomatic (n=14), mild (n=12), moderate (n=34), severe (n=18), and convalescent (n=33). Healthy controls (n = 20) came from the Shunde Hospital of Guangzhou University of Chinese Medicine. To further investigate the relative levels of factors at different points in the progression of the disease, we tracked 15 of the COVID-19 patients across three different severity groups (moderate n = 10, severe n = 4, and critical n = 1) and observed their statuses at three time points (admission to the hospital, remission of symptoms, and discharge from the hospital). This study was approved by the Ethics Committee of Shunde Hospital, Guangzhou University of Chinese Medicine (approval number: KY2020001).

### Classification of Disease Severity

Following the Protocol on Prevention and Control of Novel Coronavirus Pneumonia (Edition 6) (23), asymptomatic cases are those that have a positive COVID-19 RT-PCR test or specific serum IgM antibodies, but do not know that they are infected or show clinically identifiable symptoms. In addition, based on the Guidelines for the Diagnosis and Treatment Protocol for Novel Coronavirus Pneumonia (Trial Version 7) (24), patients with mild cases displayed mild clinical symptoms with no radiological findings of pneumonia. Moderate cases are characterized by fever (temperature above 37.3°C) and respiratory symptoms with radiological findings of pneumonia. Severe cases are those that meet any of the following criteria: respiratory distress (≥ 30 breaths/min), oxygen saturation ≤ 93% at rest, or arterial partial pressure of oxygen (PaO_2_)/fraction of inspired oxygen (FiO_2_) ≤ 300 mmHg (l mmHg = 0.133 kPa). Cases with chest imaging showing obvious lesion progression within 24–48 hours of > 50% were also managed as severe cases. Convalescent patients are considered as those with a body temperature back to normal for more than 3 days, obviously improved respiratory symptoms, obvious absorption of inflammation on pulmonary imaging, and two consecutive negative nucleic acid tests with respiratory tract samples such as sputum or nasopharyngeal swabs (sampling interval being at least 24 hours). Those who met the above criteria were eligible for being discharged.

To investigate the dynamic progression of treatment, admission, remission, and discharge timepoints were observed. The admission timepoint includes admission to the hospital within 24 hours. The remission timepoint refers to when the patient’s lung CT images have improved, and symptoms have been alleviated after treatment. The discharge timepoint refers to having a negative nucleic acid (RT-PCR) test and meeting the criterion of Diagnosis and Treatment Protocol for Novel Coronavirus Pneumonia (Trial Version 7) for discharge.

### Data Collection

The sex, age, epidemiological history, symptoms and other clinical characteristics of each patient were obtained from the electronic case system of the corresponding hospital. Routine laboratory parameters, including complete blood count, hemoglobin (Hb), C-reaction protein (CRP) and liver function data, were obtained from EDTA-anticoagulated whole blood. Sera were used for liver function, the Q440 factor antibody array, and enzyme-linked immunosorbent assay (ELISA) validation.

### Profiling With the Antibody Array

Serum levels of 440 factors were examined using antibody array, the Human Cytokine Array Q440 (QAH-CAA-440; Raybiotech, Inc., Norcross, GA, USA) was used, and each sample was assayed four times. The array included cytokines, chemokines, growth factors, and secreted enzymes, The analysis was performed strictly following the manufacturer’s protocol. The array was dried at room temperature for 2 hours, and 50 μL of serum was then placed on the array and incubated overnight at 4°C. After washing the array, 80 μL of secondary antibody solution was added to the array and incubated for 2 hours at room temperature. The array was washed again, and 80 μL of detection solution was added to the array and incubated for 1 hour at room temperature. Finally, an InnoScan 310 microarray scanner (Innopsys, Carbonne, Midi-Pyrenees, France) was used to scan the microarray and extract the signal. RayBio Q Analyzer software (Raybiotech, Inc., Norcross, GA, USA) was used to convert these fluorescence data to concentration values.

### ELISA Validation

To validate the results from the antibody array Q440, the expression levels of IL-10, IP-10, GDF15, eotaxin-2 and MCP-2 were measured using ELISA (Raybiotech, Inc., Norcross, GA, USA). The experiment was carried out according to the manufacturer’s instructions. Briefly, 100 μL of diluted serum was added into each well and incubated for 2.5 hours at room temperature. After washing the wells, 100 µL of solution containing the biotin-labeled antibody was added to each well and incubated for 1 hour at room temperature, followed by incubation of the resulting solution with a Horseradish Peroxidase (HRP)-streptavidin solution for 45 minutes at room temperature. The signal was developed using TMB One-Step Substrate Reagent which was incubated for 30 minutes at room temperature. Next, 50 μL of a stop solution was added and the absorbance at a wavelength of 450 nm was immediately recorded using an Elx800 microplate reader (BioTek Instruments, Inc., headquartered in Winooski, VT, USA). Analytical validation was performed using reference controls and biological replicates.

### Statistical Analysis

Categorical variables (demographics and baseline characteristics of patients) were described as frequency rates and percentages, and continuous variables (laboratory parameters) were described using mean, median, and interquartile range (IQR) values. No imputation was made for missing data. Clinical characteristics of COVID-19 patients were analyzed using SPSS 23.0 (IBM Corporation, Armonk, NY, USA). The Mann-Whitney test or the Kruskal-Wallis H test were used to compare the differences among the groups, and the differences were considered statistically significant at *P* < 0.05.

The R software package limma from R/Bioconductor was used to analyze differentially expressed factors associated with disease severity in COVID-19 patients. This software was specifically used to compare the levels of factors between two different groups using moderated t-statistics. The results included (log2) fold changes and *P*-values for each factor. Next, FDR (the BH method) was used to calculate adjusted *P*-values (adj.*P*.Val). Differentially expressed factors were defined as those with adjusted *P* value (adj.*P*.Val) < 0.05, and a fold-change greater than 1.2 or less than 0.83 (absolute logFC > 0.263). A Venn diagram was made online to screen for and show the logical connections between the differentially expressed factors between groups (http://bioinformatics.psb.ugent.be/webtools/Venn/).

The R software package limma was also used to analyze the dynamic changes of differentially expressed factors during progression in COVID-19 patients, using a linear empirical Bayesian model. Intra-individual correlations were included in the analysis. Differential expression was determined as an adjusted *P* value (adj.*P*.Val) < 0.10. R software (version 3.6.3; R Foundation for Statistical Computing, Vienna, Austria) was used to statistically analyze all of the differential factor data.

The receiver operating characteristic (ROC) curve was constructed using GraphPad Prism^®^ version 8.4.2 (GraphPad Software™, San Diego, CA, USA) to evaluate the diagnostic values of factors at different time points during COVID-19 disease progression. All corresponding figures were also constructed using this software.

Heat map analysis and correlation analysis (R package “gplots”), Kyoto Encyclopedia of Genes and Genomes (KEGG) enrichment analysis (R package “clusterProfiler”) and Gene Ontology (GO) enrichment analysis (R package “org.Hs. eg.db” and “clusterProfiler”) were executed using the open source program R (version 3.6.3).

## Results

### Demographics, Symptoms, and Comorbidities

Demographic and health condition data for the participants are summarized in **Table 1**. The median age of the non-severe group (asymptomatic, mild, and moderate) was 36 years (IQR, [24–49]). The median age of the asymptomatic group was 25 years (IQR, [23–45]), that of the mild group was 25 years (IQR, [22–41]), and that of the moderate group was 44 years (IQR, [28–58]). The median age of the severe group was 60 years (IQR, [55–72]), which was significantly increased compared with the non-severe group (*P* < 0.05). Further, 28% (17/60) of the patients in the non-severe group were male, whereas 56% (10/18) of the patients in the severe group were male, which was significantly increased compared with the non-severe groups (*P* < 0.05). During the course of the disease, coughing (13/18, 72%), fatigue as described by the patients (9/18, 50%), and fever (9/18, 50%) were the three most common three symptoms in the severe group, and the incidence of these symptoms were significantly increased in the severe group compared with in the non-severe groups (*P* < 0.05). Of the 111 patients with COVID-19, 77% (73/111) had one or more comorbidities, the most common of which were hypertension (32/111, 29%), liver cysts (20/111, 18%), and anemia (15/111, 14%). High blood pressure was the most common comorbidity in the severe group (9/18, 50%), followed by diabetes (4/18, 22%), and these occurrences significantly increased compared to occurrences in the non-severe groups (*P* < 0.05).

**Table 1:** Demographics and baseline characteristics of patients infected with SARS-CoV-2.

No. (%)								
	Healthy Control	Non-Severe				Non-Severe *vs* Severe	Severe	Convalescent
		Asymptomatic	Mild	Moderate	Total Non-Severe			
	(N = 20)	(N = 14)	(N = 12)	(N = 34)	(N = 60)	*P* value	(N = 18)	(N = 33)
**Age, median (IQR), y**	38 (30–47)	25 (23–45)	25 (22–41)	44 (28–58)	36 (24–49)	< 0.0001	60 (55–72)^a^**^b^**^c^**^d*^	62 (40-69)^a^*^b^**^c*^
**Sex**						0.034		
Male	12 (60%)	4 (29%)	4 (33%)	9 (27%)	17 (28%)		10 (56%)	12 (36%)
Female	8 (40%)	10 (71%)	8 (67%)	25 (74%)	43 (72%)	8 (44%)	21 (64%)	
**Symptom**								
Cough	0	1 (7%)	7 (58%)^a*^	19 (56%)^a**^b^*^	27 (45%)	0.044	13 (72%)^a** b**^	16 (49%)^a **^
Expectoration	0	1 (7%)	4 (33%)^a*^	11 (32%)^a**^	16 (27%)	0.707	4 (22%)	10 (30%)^a*^
Fatigue	0	0	3 (25%)	8 (24%)	11 (18%)	0.007	9 (50%)^a**^b^*^	10 (30%)
Chills	0	0	0	9 (27%)	9 (15%)	0.008	8 (44%)^a*^b^*^c^*^	9 (27%)
Sore throat	0	2 (14%)	4 (33%)	7 (21%)	13 (22%)	0.96	4 (22%)	8 (24%)
Fever	0	0	2 (17%)	12 (35%)^a*^	14 (23%)	0.013	9 (50%)^a**^b^*^	0^d** e**^
Shortness of breath	0	0	0	4 (12%)	4 (7%)	0.003	6 (33%)^a*^	7 (21%)
Headache	0	0	2 (17%)	6 (18%)	8 (13%)	0.362	4 (22%)	4 (12%)
Myalgia	0	0	1 (8%)	3 (9%)	4 (7%)	0.058	4 (22%)	4 (12%)
Chest tightness	0	0	0	2 (6%)	2 (3%)	0.009	4 (22%)	5 (15%)
Rhinorrhea	0	0	1 (8%)	7 (21%)	8 (13%)	0.368	1 (6%)	2 (6%)
Diarrhea	0	0	1 (8%)	3 (9%)	4 (7%)	0.058	4 (22%)	2 (6%)
Nausea and vomiting	0	0	0	4 (12%)	4 (7%)	0.537	2 (11%)	2 (6%)
Dry throat	0	1 (7%)	5 (42%)^b**^	0^c**^	6 (10%)	0.892	2 (11%)^c*^	0^c**^
**Comorbidity**								
Hypertension	0	0	3 (25%)	7 (21%)	10 (17%)	0.004	9 (50%)^a**^b^*^	13 (39%)^a*^
Liver cyst	0	1 (7%)	1 (8%)	6 (18%)	8 (13%)	0.806	2 (11%)	11 (33%)^a*^
Anemia	0	1 (7%)	1 (8%)	7 (21%)	9 (15%)	0.68	2 (11%)	4 (12%)
Cardiovascular disease	0	0	1 (8%)	3 (9%)	4 (7%)	0.537	2 (11%)	5 (15%)
Hyperlipemia	0	0	1 (8%)	3 (9%)	4 (7%)	2 (11%)	4 (12%)	
Diabetes	0	0	1 (8%)	2 (6%)	3 (5%)	0.026	4 (22%)	1 (3%)
Respiratory disease		2 (14%)	0	1 (3%)	1 (2%)	0.069	2 (11%)	5 (15%)
Cerebrovascular **Disease**	0	0	0	2 (6%)	2 (3%)	0.192	2 (11%)	4 (12%)
Hyperuricemia	0	0	0	3 (9%)	3 (5%)	0.356	2 (11%)	2 (6%)
Fatty liver	0	0	0	2 (6%)	2 (3%)	0.192	2 (11%)	2 (6%)
Hepatitis B	0	0	0	2 (6%)	2 (3%)	0.669	1 (6%)	3 (9%)
Hypertriglyceridemia	0	2 (14%)	0	2 (6%)	4 (7%)	0.264	0	0
Kidney stone	0	0	0	2 (6%)	2 (3%)	0.436	0	1 (3%)

1. The difference showed with: ^a^Compared with healthy controls; ^b^Compared with the asymptomatic group; ^c^Compared with the mild group; ^d^Compared with the moderate group; ^e^Compared with the severe group. *P < 0.05, **P < 0.01. IQR, interquartile range; Clinical features are listed in order of frequency.

2. In the asymptomatic group, there were two patients with symptoms of chronic coughing and expectoration due to chronic respiratory disease (one with chronic bronchitis and the other with chronic pharyngitis), but there were no symptoms associated with COVID-19, such as low fever and fatigue, after a positive nucleic acid SARS-CoV-2test, so the patients were considered to be in the asymptomatic group.

### Laboratory Characteristics of COVID-19 Patients

**Figure 1:**
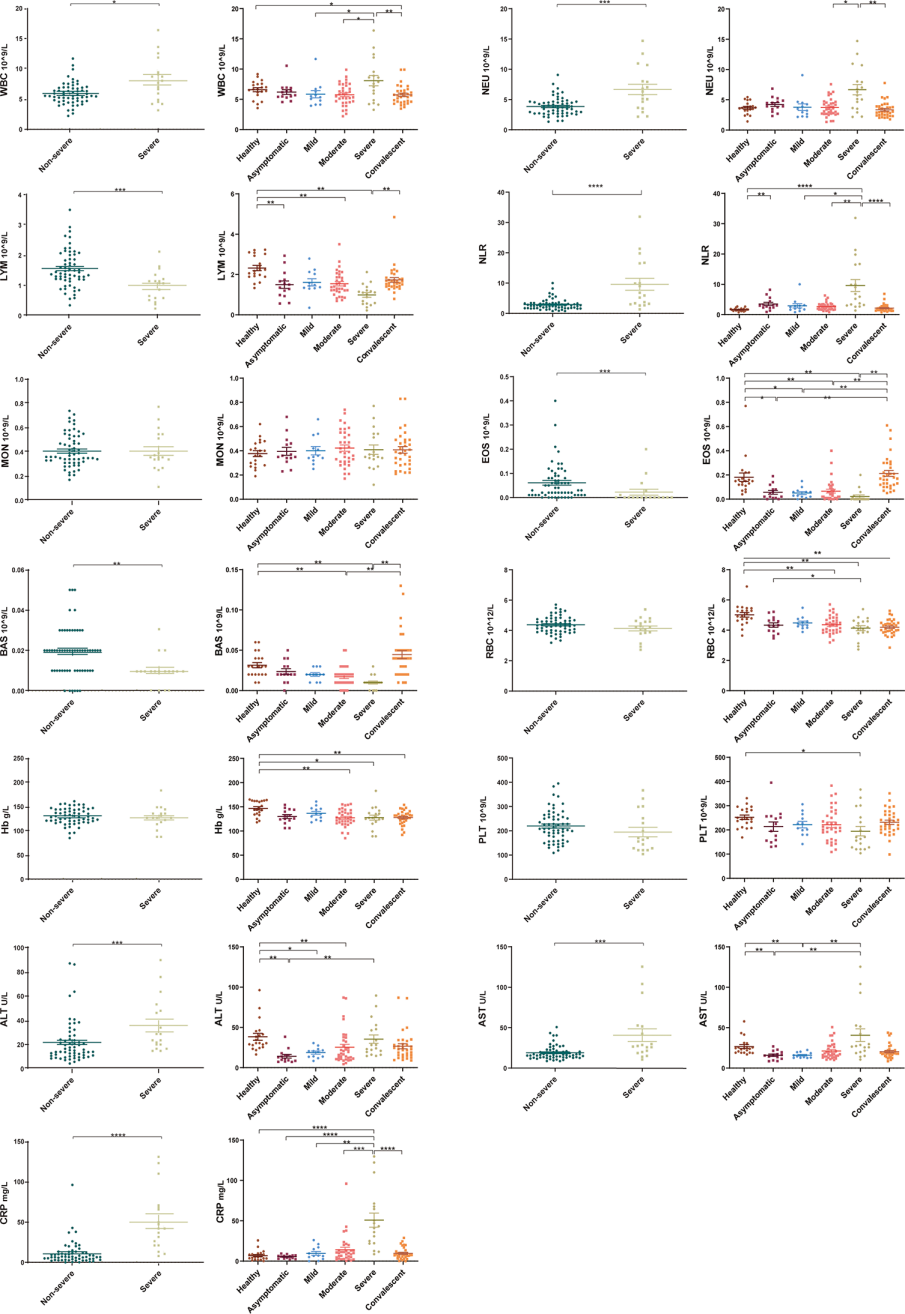
Laboratory parameters from different COVID-19 severity groups (asymptomatic, mild, moderate, severe and convalescent) and healthy controls. Differential levels of routine complete blood count, hemoglobin (Hb), C-reaction protein (CRP) and liver function across different severity groups. White blood cell count, neutrophil count, neutrophil-lymphocyte ratio (NLR), alanine aminotransferase and aspartate aminotransferase levels were higher in the severe group compared with the non-severe groups (asymptomatic, mild, and moderate) (*P* < 0.05). Lymphocyte and eosinophil counts were significantly decreased in the severe group compared with the non-severe group (*P* < 0.001), with a trend to recovery in the convalescent group. Healthy control (n=20), asymptomatic group (n=14), mild group (n=12), moderate group (n=34), severe group (n=18), non-severe group (n=60), convalescent group (n=33). Significance is denoted as *P < 0.05, **P < 0.01, ***P < 0.001, and ****P < 0.0001. WBC, White blood cell count; NEU, Neutrophils count; LYM, Lymphocytes count; NLR, neutrophil-lymphocyte ratio; MON, Monocyte count; EOS, Eosinophil count; BAS, Basophils count; RBC, Erythrocyte count; Hb, Haemoglobin; PLT, Platelet count; ALT, Alanine aminotransferase; AST, Aspartate aminotransferase; CRP, C-reactive protein.

**Figure 2:**
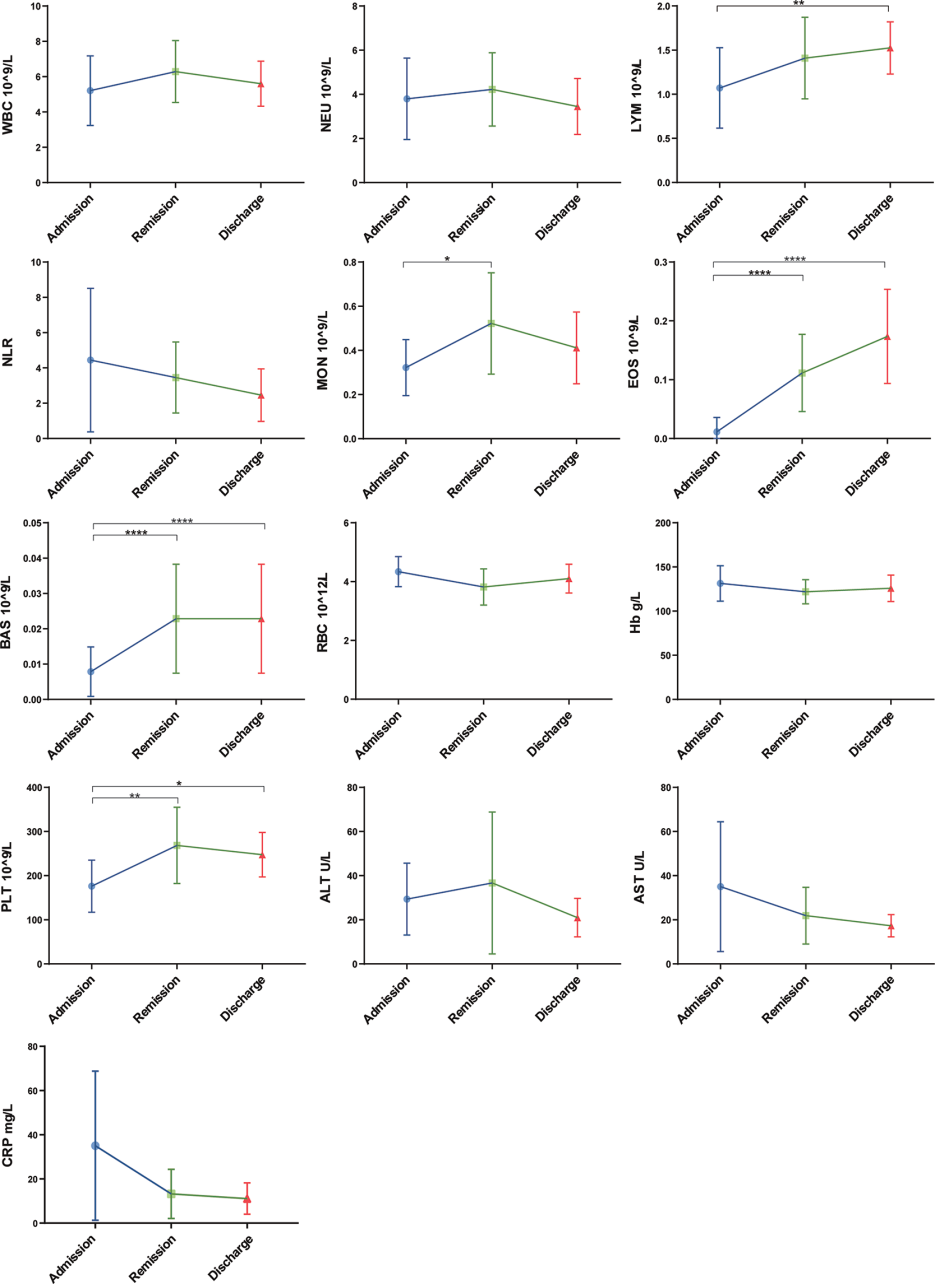
Laboratory parameters with the progression of COVID-19/patient status (at three time points: admission, remission, and discharge). Different levels of routine complete blood count, hemoglobin (Hb), C-reaction protein (CRP) and liver function were found at different time-points of disease/patient status progression. Moreover, as patients improved, lymphocyte and eosinophil counts consistently increased, whereas NLR and AST consistently decreased. Admission group (n=14), remission group (n=14), discharge group (n=14). Significance is denoted as *P < 0.05, **P < 0.01 and ****P < 0.0001. WBC, White blood cell count; NEU, Neutrophils count; LYM, Lymphocytes count; NLR, neutrophil-lymphocyte ratio; MON, Monocyte count; EOS, Eosinophil count; BAS, Basophils count; RBC, Erythrocyte count; Hb, Haemoglobin; PLT, Platelet count; ALT, Alanine aminotransferase; AST, Aspartate aminotransferase; CRP, C-reactive protein.

The routine clinical laboratory characteristics of the participants are shown in **Table 2** and **Figure 1**. In the severe group, the median white blood cell count was 7.74×10^9^/L (IQR, [4.73–10.14]) and the median neutrophil count was 6.02×10^9^/L (IQR, [3.61–8.70]), which was significantly increased compared to the median white blood cell count was 5.60×10^9^/L (IQR, [4.69–6.86]) and the median neutrophil count was 3.72×10^9^/L (IQR, [2.71–4.62]) in the non-severe group (*P* < 0.05). The median lymphocyte counts in the non-severe group and severe group were 1.42×10^9^/L (IQR, [1.20–1.98]) and 1.08×10^9^/L (IQR, [0.56–1.25]), respectively, whereas the median eosinophil counts in the non-severe group and severe group were 0.04×10^9^/L (IQR, [0.01–0.09]) and 0.00×10^9^/L (IQR, [0–0.015]), respectively. Both lymphocytes and eosinophils were significantly decreased in the severe group compared with the counts in the non-severe group (*P* < 0.001). We found a trend in the lymphocyte and eosinophil counts in the convalescent group to recovery. The lymphocyte count decreased with increasing disease severity in the mild, moderate and severe groups. The neutrophil-lymphocyte ratio (NLR) was 6.38×10^9^/L (IQR, [3.15–16.55]) in the severe group, which was significantly increased compared with the non-severe group (2.59×10^9^/L, IQR, [1.73-3.38], *P* < 0.001). Alanine aminotransferase (ALT) levels were 26.10 U/L (IQR, [18.75-49.08]) in the severe group and 15.25 U/L (IQR, [10.75–24.29]) in the non-severe group. Aspartate aminotransferase (AST) levels were 26.95 U/L (IQR, [19.48–51.73]) in the severe group and 16.25 (IQR, [13.15–21.25]) in the non-severe group. Both ALT and AST levels were significantly increased in the severe group compared with the non-severe group (*P* < 0.001). The C-reactive protein (CRP) expression in the severe group was 42.03 mg/L (IQR, [22.06–68.63]), and was significantly lower in the non-severe group (6.79 mg/L, IQR, [3.37–12.88], *P* < 0.001). Moreover, we found that the lymphocyte, eosinophil, and platelet counts continuously increased with disease progression and patient status (admission, remission, and discharge), as shown in **Table 3** and **Figure 2**.

**Table 2:** Laboratory parameters of SARS-CoV-2-infected patients classified by disease severity.

Median (IQR)								
	Healthy Control	Non-Severe					Severe	Convalescent
		Asymptomatic	Mild	Moderate	Total non-severe	Non-Severe *vs*. Severe		
	(N = 20)	(N = 14)	(N = 12)	(N = 34)	(N = 60)	*P* value	(N=18)	(N = 33)
**White blood cell count** × 10^9^/L	6.66 (5.83–7.56)	5.93 (5.40–6.88)	5.43 (4.30–6.61)	5.68 (4.50–7.33)	5.60 (4.69–6.86)	0.014	7.74 (4.73–10.14)^c*^d^*^	5.65 (4.62-6.44)^a*^e^**^
**Neutrophil count** × 10^9^/L	3.66 (3.23–4.26)	4.20 (3.36–4.95)	3.35 (2.35–4.44)	3.44 (2.69–4.57)	3.72 (2.71–4.62)	0.0007	6.02 (3.61–8.70)^d*^	2.98 (2.45-3.90)^e**^
**Lymphocyte count** × 10^9^/L	2.21 (1.90–3.00)	1.43 (0.97–1.83)^a**^	1.46 (1.33–2.11)	1.39 (1.17–1.91)^a**^	1.42 (1.20–1.98)	0.0003	1.08 (0.56–1.25)^a**^	1.59 (1.42-1.82)^e**^
**Neutrophil-lymphocyte ratio**	1.59 (1.28–1.90)	3.02 (2.31–4.67)	2.28 (1.64–3.08)	2.52 (1.71–3.41)	2.59 (1.73–3.38)	< 0.0001	6.38 (3.15–16.55)	1.72 (1.41-2.56)
**Monocyte count** × 10^9^/L	0.38 (0.28–0.47)	0.36 (0.33–0.47)	0.36 (0.33–0.50)	0.39 (0.31–0.55)	0.37 (0.31–0.53)	0.9133	0.36 (0.32–0.54)	0.39 (0.29-0.49)
**Eosinophil count** × 10⁹/L	0.15 (0.11–0.21)	0.04 (0.01–0.09)^a*^	0.05 (0.02–0.07)^a*^	0.04 (0.01–0.10)^a**^	0.04 (0.01–0.09)	0.0002	0.00 (0–0.015)^a**^	0.17 (0.12-0.30)^b**^c^**^d^**^e^**^
**Basophil count** × 10^9^/L	0.03 (0.02–0.04)	0.02 (0.02–0.03)	0.02 (0.01–0.03)	0.02 (0.01–0.02)^a**^	0.02 (0.01–0.03)	0.0012	0.01 (0.01–0.01)^a**^	0.04 (0.02-0.05)^d**^e^**^
**Erythrocyte count** × 10^12^/L	5.02 (4.66–5.41)	4.32 (3.90–4.71)^e*^	4.39 (4.19–4.70)	4.32 (3.91–4.81)^a**^	4.33 (3.97–4.73)	0.2792	4.19 (3.84–4.60)^a**^	4.16 (3.88-4.48)^a**^
**Hemoglobin** g/L	145.50 (134.50–162.00)	128.50 (120.50–144.25)	135.00 (124.25–146.75)	125.00 (116.75–140.75)^a**^	128.50 (120.00–144.00)	0.7309	127.00 (118.00–138.25)^a*^	129.00 (120.50-135.50)^a**^
**Platelet count** × 10^9^/L	246.00 (215.50–286.00)	210.00 (154.25–256.25)	217.00 (184.75–260.50)	208.50 (160.75–270.00)	208.50 (165.50–257.75)	0.0906	166.50 (126.00–263.75)^a*^	227.00 (200.50-265.50)
**Alanine aminotransferase** U/L	30.60 (26.23–44.33)	12.00 (8.33–16.03)^a**^	20.15 (13.58–22.70)^a*^	17.00 (10.85–34.78)^a**^	15.25 (10.75–24.29)	0.0008	26.10 (18.75–49.08)^b**^	22.90 (15.15-31.20)
**Aspartate aminotransferase** U/L	24.10 (19.03–29.55)	15.85 (11.00–20.05)^a**^	15.10 (12.88–19.38)^a**^	17.10 (14.05–27.25)	16.25 (13.15–21.25)	0.0003	26.95 (19.48–51.73)^b**^c^**^	18.20 (15.60-22.10)
**C-reactive protein** mg/L	4.88 (2.28–9.32)	5.87 (3.12–7.07)	6.73 (3.25–16.47)	9.98 (4.15–15.00)	6.79 (3.37-12.88)	< 0.0001	42.03 (22.06–68.63)^b^**^c^**^d^**	7.78 (5.21-12.69)^e^**

The difference showed with: ^a^Compared with healthy controls; ^b^Compared with the asymptomatic group; ^c^Compared with the mild group; ^d^Compared with the moderate group; ^e^Compared with the severe group. *P < 0.05, **P < 0.01. IQR, interquartile range.

**Table 3:** Laboratory parameters of SARS-CoV-2-infected patients classified by disease/patient status progression.

Median (IQR)				
	Admission	Remission	Discharge	
	(N = 14)	(N = 14)	(N = 14)	*P* value
**White blood cell count** × 10^9^/L	4.76 (3.90–6.68)	6.03 (5.09–7.65)	5.52 (4.58–6.66)	0.3218
**Neutrophil count** × 10^9^/L	3.49 (2.68–4.81)	4.18 (2.45–5.79)	3.27 (2.40–4.55)	0.3642
**Lymphocyte count** × 10^9^/L	1.13 (0.79–1.29)	1.27 (1.08–1.71)	1.56 (1.38–1.71)^a**^	0.005
**Neutrophil-lymphocyte ratio**	3.23 (1.94–5.01)	2.76 (1.57–5.40)	1.94(1.44–3.08)	0.1075
**Monocyte count** × 10^9^/L	0.32 (0.21–0.40)	0.42 (0.34–0.65)^a*^	0.40 (0.28–0.50)	0.0241
**Eosinophil count** × 10⁹/L	0 (0–0.01)	0.09 (0.07–0.18)^a****^	0.17 (0.11–0.23)^a****^	<0.0001
**Basophil count** × 10^9^/L	0.01 (0.00–0.01)	0.02 (0.01–0.03)^a****^	0.04 (0.02-0.05)^a****^	0.0019
**Erythrocyte count** × 10^12^/L	4.32 (3.99–4.68)	3.97 (3.59–4.20)	4.12 (3.85–4.47)	0.0893
**Hemoglobin** g/L	129.00 (120.50–139.00)	121.00 (115.00–129.50)	126.00 (120.25–132.25)	0.2229
**Platelet count** × 10^9^/L	160.00 (134.75–200.75)	266.50 (197.25–341.75)^a**^	244.00 (211.00–281.25)^a*^	0.002
**Alanine aminotransferase** U/L	23.55 (16.63–42.85)	26.75 (20.48–44.45)	22.35 (12.38-27.28)	0.2068
**Aspartate aminotransferase** U/L	27.25 (15.13–45.15)	19.80 (15.05–22.95)	17.85 (12.98–19.78)	0.0811
**C-reactive protein** mg/L	33.86 (4.14–47.78)	7.76 (5.94–22.29)	8.14 (6.22–19.60)	0.3218

^a^Compared with admission. *P < 0.05, **P < 0.01 and ****P < 0.0001. IQR, interquartile range.

### Factor Profiling With Antibody Arrays

**Figure 3:**
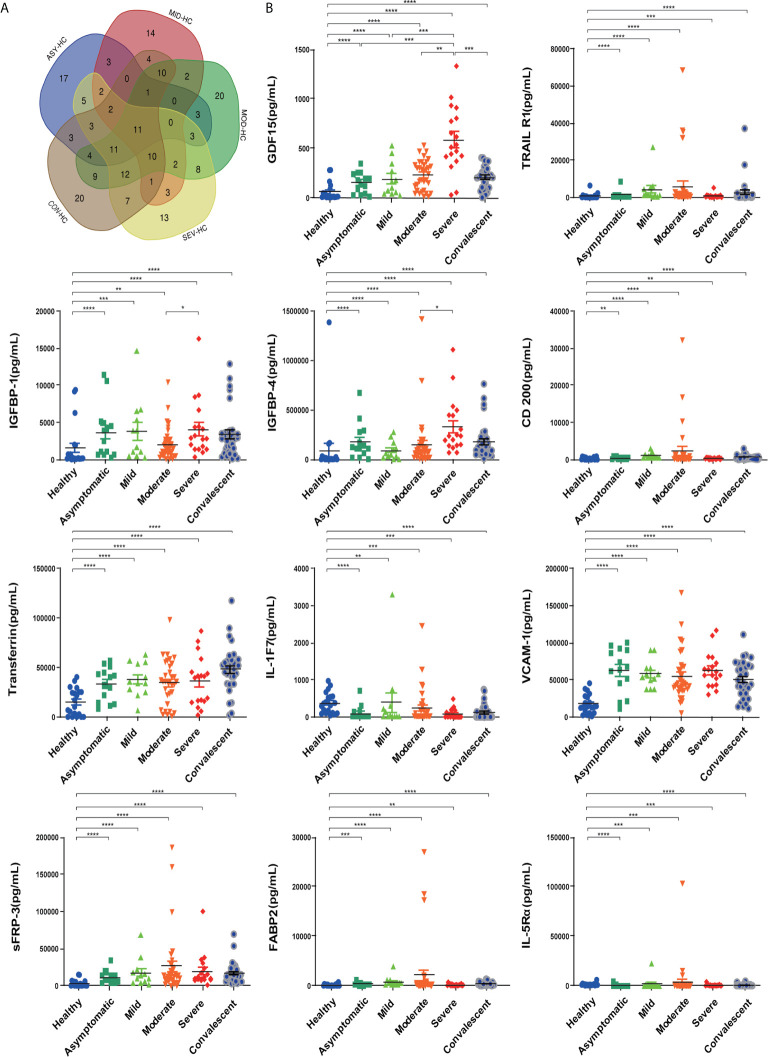
Eleven differentially expressed factors were highly associated with COVID-19 disease severity. **(A)** Venn diagram displaying the common differentially expressed cytokines in patients with COVID-19 of different severities. **(B)** Individual levels of the eleven differentially expressed cytokines in different COVID-19 severity groups. Healthy control (n=20), asymptomatic group (n=14), mild group (n=12), moderate group (n=34), severe group (n=18), convalescent group (n=33). Significance is denoted as *P < 0.05, **P < 0.01, ***P < 0.001, and ****P < 0.0001. HC, Healthy Control; ASY, Asymptomatic; MIL, Mild; MOD, Moderate; SEV, Severe; CON, Convalescent.

To investigate the factors associated with COVID-19, we first used an antibody array to screen 440 factors in sera from different disease severity groups (asymptomatic, mild, moderate, severe and convalescent) and with healthy controls. Differentially expressed factors from the 440 factors assessed in each of the different disease severity groups compared to the healthy control group were determined under the screening conditions of adj.*P*.Val < 0.05 and fold-change greater than 1.2 or less than 0.83 (absolute logFC > 0.263).There were 68 differential factors identified in the asymptomatic group, 65 differential factors identified in the mild group, 106 differential factors identified in the moderate group, 90 differential factors identified in the severe group, and 108 differential factors identified in the convalescent group. We further analyzed and compared taxon overlap among these intergroup differential factors using Venn diagrams (**Figure 3A**). Finally, a total of eleven differential factors related to COVID-19 disease severity were identified, including: TRAIL R1, IGFBP-1, IGFBP-4, VCAM-1, sFRP-3, FABP2, Transferrin, GDF15, IL-1F7 (also known as IL-37), IL-5Rα, and CD200. 9 of the 11 differentially expressed factors were significantly increased in COVID-19 patients compared with healthy controls: TRAIL R1, IGFBP-1, IGFBP-4, VCAM-1, sFRP-3, FABP2, Transferrin, GDF15, and CD200 (*P* < 0.01). 2 of the 11 differentially expressed factors: IL-1F7 and IL-5Rα were significantly decreased in COVID-19 patients compared with healthy controls (*P* < 0.01) (**Figure 3B**). In addition, we found a consistent increase in GDF15 levels with increasing disease severity among the asymptomatic, mild, moderate, and severe groups of COVID-19 patients. Moreover, GDF15 expression in the convalescent group was recovered to similar levels compared to the healthy control group, suggesting that GDF15 levels closely reflected the progression of the disease.

To investigate possible independent predictors for the progression of COVID-19, we further analyzed the eleven differentially expressed COVID-19-associated factors over the disease/treatment progression at 3 time points: admission, remission and discharge. Although most of the eleven factors showed dramatic differences in expression between the groups with different disease severity, the correlation between the magnitude of the changes and disease progression was not consistent.

**Figure 4:**
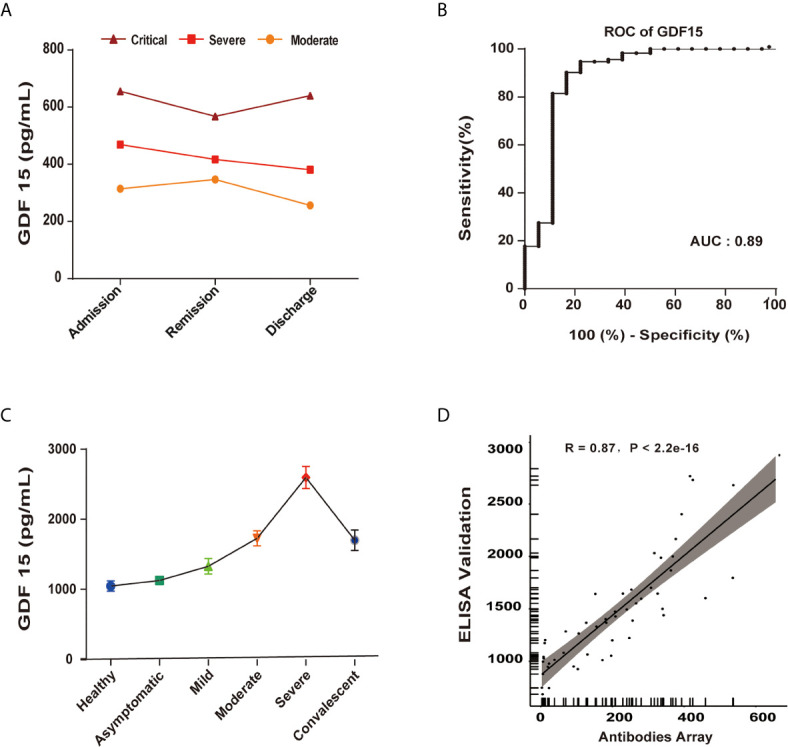
GDF15 is an independent predictor of the progression of COVID-19. **(A)** GDF15 levels in critical (n=1), severe (n=4), and moderate (n=10) groups, each as a function of the progress of the infection/patient status. **(B)** The AUC value of 0.89 indicated the serum GDF15 level to be an efficient diagnostic indicator of disease severity in COVID-19 patients. **(C)** ELISA-determined GDF15 level as a function of disease severity. **(D)** ELISA-determined GDF15 levels compared to antibody array-determined levels showed a strong correlation (R = 0.87, *P* < 2.2e−16).

To further validate GDF15 as an independent predictor of the severity of COVID-19, the correlation between GDF15 levels and disease/treatment stage (admission, remission, and discharge) was analyzed. In the moderate group, GDF15 levels increased mildly from admission to remission, indicating that the disease was progressing. However, GDF15 levels decreased markedly from remission to discharge. In the severe group, the levels of GDF15 gradually decreased with recovery progress across admission, remission, and discharge (**Figure 4A**). Taken together, the changes over time in GDF15 levels reflected the progression of the disease. The diagnostic value of GDF15 was evaluated using area under the curve (AUC) analysis by the receiver operating characteristic (ROC) curve. The AUC value of 0.89 (**Figure 4B**) indicated the serum GDF15 level to be an efficient diagnostic indicator of disease severity in COVID-19 patients.

To validate the results of the antibody array profile, we used ELISA to measure the levels of IL-10, IP-10, GDF15, eotaxin-2 and MCP-2. We found the results of the antibody array to be well correlated with the ELISA results, indicating the data were reliable. The GDF15 expression as determined by ELISA showed a strong correlation with the expression as determined by antibody array (R = 0.87, *P* < 2.2e−16) (**Figures 4C, D**).

GO enrichment analysis included biological process (BP), cell component (CC), and molecular function (MF) terms. As displayed in **Supplementary Figure 2A**, CCs including GO: 0005576 (extracellular region) and GO: 0005615 (extracellular space) were significantly associated with differentiation. KEGG pathways with *P* < 0.05 are shown in **Supplementary Figure 2B**. Including hsa04061 (Viral protein interaction with cytokine and cytokine receptor) and hsa04060 (Cytokine-cytokine receptor interaction) were significantly associated with the identified differentially expressed factors.

## Discussion

As the number of COVID-19 patients continues increasing dramatically worldwide, the treatment of severe cases has become a major challenge. Consequently, the early recognition of severe forms of COVID-19 is essential for achieving a timely triaging of patients.

In a cohort of 111 patients who tested positive for COVID-19, studied here, we found that both the average age and the proportion of males was increased in the severe group compared with the non-severe group, which is consistent with the general recognition of age being an important risk factor for developing severe COVID-19 (25). In our cohort, 65.77% of the patients had at least one comorbidity. High blood pressure was the most common comorbidity in the severe group, followed by diabetes, which is consistent with previous reports suggesting that SARS-CoV-2 is more likely to infect people with chronic comorbidities due to impaired immune function (26, 27). Most interestingly, other comorbidities including liver cysts, anemia, cardiovascular disease and high blood lipid levels were observed in our study, which, to the best of our knowledge, have not been previously reported.

Studies have reported a significant increase in white blood cell (WBC) count in patients with severe COVID-19 that is associated with poor prognosis. This phenomenon is consistent with the findings in our study. The increase in WBC counts in severely affected patients may have been driven by elevated neutrophil levels, as well as decreased levels of lymphocytes and eosinophils (28, 29). Several studies have reported that a decrease in lymphocytes and eosinophils was the most commonly found clinical characteristics of patients with COVID-19 (30). In the current study, the lymphocyte and eosinophil counts decreased gradually with increasing disease severity, but then increased at the convalescent stage. The observed increased NLR (i.e. the combination of a decrease in lymphocyte levels and an increase in neutrophil levels) is a hallmark of inflammation and infection throughout the body and has been studied as a predictor of bacterial infection (31). Our study found that the NLR was increased in COVID-19 patients, most significantly in the severe group, compared with healthy controls. This was consistent with the findings of other studies (32, 33), indicating a serious interference of the internal environment of the body by the potential critical conditions of these severe infections. CRP, a routinely measured inflammatory marker, that was also observed in the current study, has previously been shown to be elevated in most patients with COVID-19 (34). In our study, CRP was significantly elevated in the severe group, and appeared to correlate with disease severity.

The cytokines IL-6 and IL-8 have been previously suggested to be associated with the severity of COVID-19 (15). Here, we found the serum levels of IL-6 and IL-8 to have increased with increasing COVID-19 severity with COVID-19 disease progression. Moreover, we identified eleven factors: TNF-related apoptosis-inducing ligand (TRAIL)-R1, IGFBP-1, IGFBP-4, VCAM-1, sFRP-3, FABP2, transferrin, GDF15, IL-1F7, IL-5Rα, and CD200 differentially expressed among asymptomatic, mild, moderate, severe, and convalescent COVID-19 patients compared with healthy controls using an antibody array analysis of 440 factors.

TRAIL at high serum concentrations has been proposed to be an important mediator of adipose tissue inflammation and obesity-associated diseases (35, 36). Here, we found much higher levels of TRAIL R1 in the 8 patients with diabetes compared to non-diabetic patients, indicating that TRAIL R1 might contribute to diabetes-related COVID-19 disease severity. Insulin-like growth factor 1 (IGF-1) has been reported to play critical roles in diverse biological activities including cell metabolism, tissue regeneration, and survival (37). IGF-binding proteins (IGFBPs) bind both insulin-like growth factors (IGFs) I and II, which can circulate in the plasma in both glycosylated and non-glycosylated forms. Binding of any of these proteins to an IGF prolongs the half-life of the IGF and alters the interaction of the IGF with cell surface receptors (38). In this study, IGFBP-1 and IGFBP-4 had the highest expression levels in the severe group, suggesting a role in organ damage and consequent activation of the wound-healing process (39). In addition, the levels of IL-5Rα, FABP2, CD200 and transferrin consistently showed the highest levels in the moderate group, indicating that these factors may serve as sensitive biomarkers reflecting COVID-19 disease severity.

Growth differentiation factor-15 (GDF15) was previously reported to be associated with inflammation, and many biological processes and diseases, including cancer and obesity (40). The GDF15 gene is located on human chromosome 19p13.1-13.2. GDF15 is synthesized as pro-GDF15, which is then dimerized, cleaved and secreted into the circulation as a mature GDF15 dimer (41). Both the intracellular GDF15 and the circulating mature GDF15 are implicated in biological processes such as energy homeostasis and body weight regulation. GDF15 is a member of the transforming growth factor β (TGF-β) superfamily and is widely expressed in a variety of mammalian tissues, where it is highly regulated and often inducibly expressed under conditions associated with cellular stress (42). GDF15 is also well known to be a stress-induced factor released in response to tissue damage (43). Zimmers et al. reported that GDF15 expression is upregulated in mouse models of kidney and lung injury (44). Hsiao et al. have also shown a significant and rapid increase in GDF15 levels following injury to liver hepatocytes or bile duct epithelial cells (45). Serum levels of GDF15 are strongly associated with many diseases, and functional roles of GDF15 have been reported in cardiovascular, renal, cancer, and metabolic diseases (40). However, knowledge of its pathophysiological function at the molecular level is still limited and more studies are needed. The recent discovery that GFRAL is the endogenous receptor for GDF15 may provide additional insight into the molecular mechanism of GDF15 and its relationship to disease states (46). A recent experimental study has shown a novel function of GDF15 in promoting rhinovirus-associated lung inflammation in humans, which can lead to severe respiratory viral infections (47). A relationship between GDF15 and COVID-19 was reported recently. Myhre et al. showed that GDF15 concentrations were not only independently associated with risk but also used to identify key pathophysiological features of COVID-19, including hypoxemia in comparison of non-ICU and ICU (or death) patients. They also provided prognostic information superior to that provided by the established risk markers in COVID-19 (48). In another study, Notz et al. similarly reported an increase in the level of GDF15 during ICU treatment, suggesting increased tissue resilience (49). Remarkably, in the current study, we found GDF15 levels to be closely associated with disease progression of COVID-19.

To further evaluate the potential of GDF15 as a biomarker of disease severity, we longitudinally collected serum samples, specifically from three groups with different disease severities (moderate, severe, and critical) at three time points (admission, remission, and discharge). The average level of GDF15 increased with severity. Specifically, GDF15 levels were 305.930 ± 85.8pg/mL in the moderate group, 422.5 ± 80.9pg/mL in the severe group, and 621pg/mL in the critical group, with the critical group having a nearly 1.47-times increase compared with the severe group, and a 1.88-times increase compared to the moderate group. In the four patients with severe symptoms, the GDF15 levels showed a linear decrease from admission to the hospital to remission of symptoms to discharge from the hospital. In the ten patients with moderate symptoms, GDF15 increased slightly from admission to remission, but dropped dramatically from remission to discharge. Coincidentally, one critical patient had very high levels of GDF15 at admission, which decreased slightly at remission. Upon worsening of symptoms prior to death, we found that the GDF15 level increased. These observations provided evidence that GDF15 might serve as an indicator of disease severity in COVID-19 patients.

Our findings provide important knowledge related to the severity stratification of COVID-19. However, due to the improvement of the epidemic situation in China, the sample size of this study was limited, especially for the critically ill patients. Our findings should be validated in a larger, more diverse population and environment.

## Data Availability Statement

The original contributions presented in the study are included in the article/**Supplementary Material**. Further inquiries can be directed to the corresponding authors.

## Ethics Statement

The studies involving human participants were reviewed and approved by the ethics committees of Shunde Hospital of Guangzhou University of Chinese Medicine. The patients/participants provided their written informed consent to participate in this study.

## Author Contributions

JHX, DWW, YLS, YTL, RPH designed the study; YLS, YXL provided samples; JYH, JQZ, YLH, DDL, YZL conducted experiments; YQY, SHL, SZZ analyzed data; XYT, JQZ participated in the manuscript writing. All authors contributed to the article and approved the submitted version.

## Funding

This study was supported by the Science and Technology Innovation Project of Foshan Municipality (2020001000431), the National Key Research and Development Project (2020YFA0708001), the National Science and Technology Major Project of China (No. 2018ZX10732-401-003-012) and the Industry-University-Research Collaborative Innovation Special Project of Guangzhou (201802030001).

## Conflict of Interest

Authors YQY, SHL, SZZ and RPH were employed by company RayBiotech Inc.

The remaining authors declare that the research was conducted in the absence of any commercial or financial relationships that could be construed as a potential conflict of interest.
