# Supplementary material for: Comprehensive Profiling of Inflammatory Factors Revealed That Growth Differentiation Factor-15 Is an Indicator of Disease Severity in COVID-19 Patients
Source: Front Immunol. 2021 Jul 15;12:662465. doi: 10.3389/fimmu.2021.662465 (PMC8320433; doi:10.3389/fimmu.2021.662465)
Supplement: Supplementary file 1 [file DataSheet_1.docx]

Supplementary Material

# Supplementary Figures and Tables

[**Supplementary**](javascript:;) **Figure 1.** Heatmap of cytokines differentially expressed in COVID-19 patients with different severities. Cytokine names are shown at the bottom of the chart. Low expression is indicated in blue and high expression in red. Different severities are highlighted by the color code on the left.

[**Supplementary**](javascript:;) **Figure 2.** Gene Ontology (GO) and Kyoto Encyclopedia of Genes and Genomes (KEGG) pathway enrichment analyses using clusterProfiler. (A) GO analysis depicting the significantly enriched GO terms for the top enriched protein sets from differentially expressed proteins. BP, Biological process; CC, Cellular component; MF, Molecular function. (B) KEGG analysis depicting the significantly enriched GO terms for the top enriched protein sets from differentially expressed proteins. The colors represent the P-values relative to the other displayed terms, with blue indicating the greatest significance. The size of each round node is proportional to the ratio of the number of proteins to the number of enriched proteins.


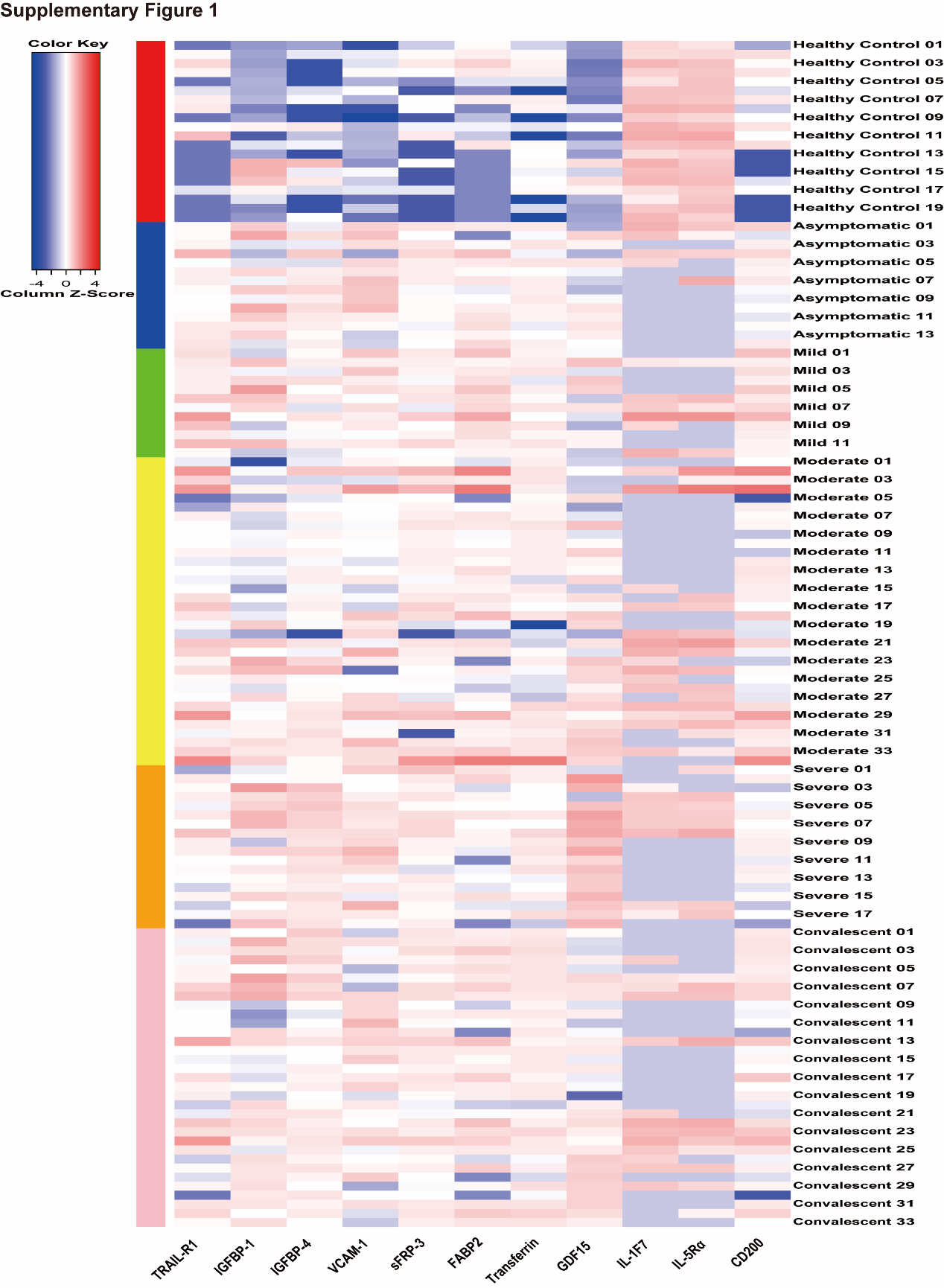


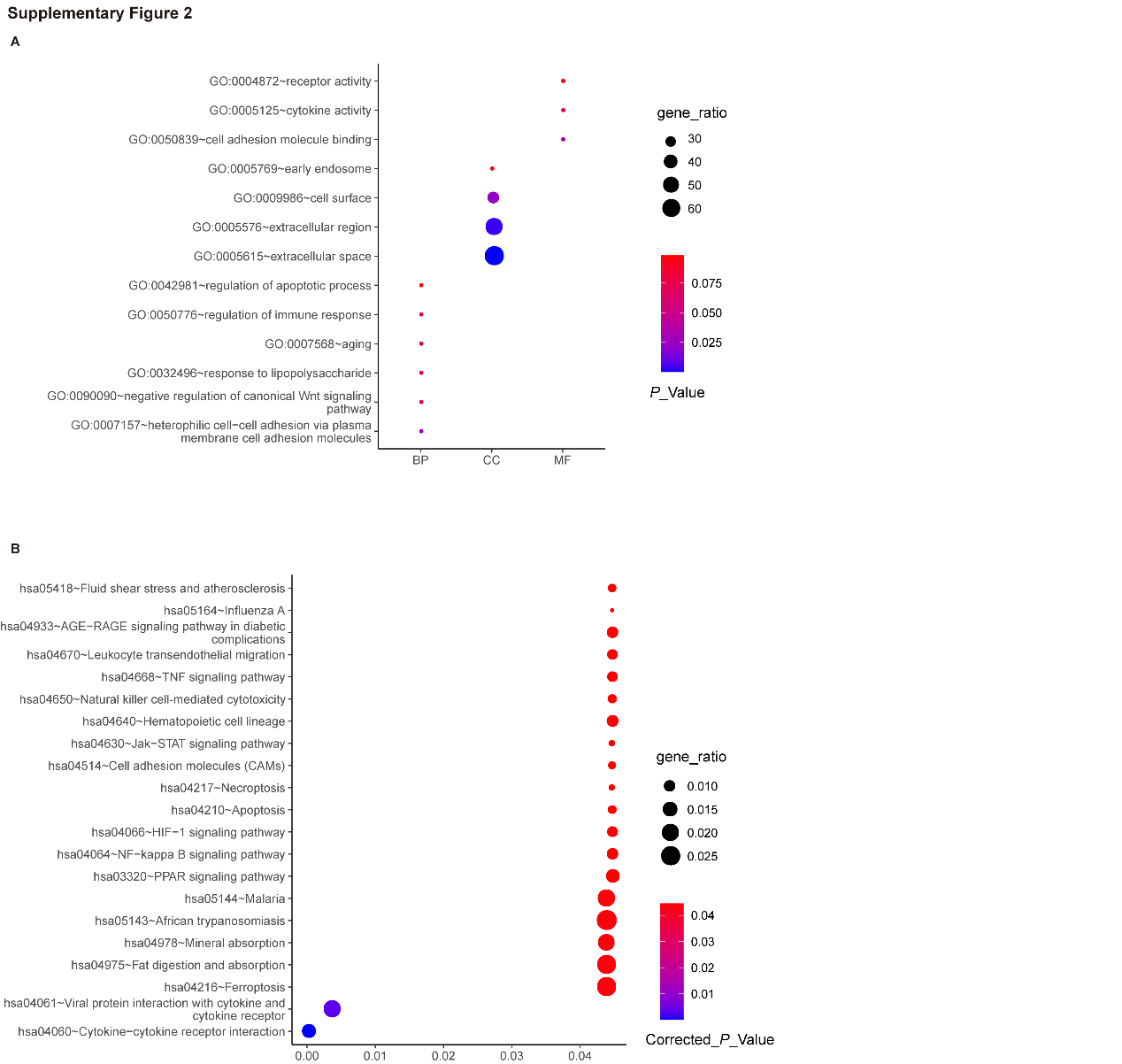


**Supplementary Table 1**

Changes in concentrations of differential factors with across the COVID-19 different severity of groups COVID-19

| **Median (IQR)** | | | | | | |
| --- | --- | --- | --- | --- | --- | --- |
|  | **Healthy Control** | **Asymptomatic** | **Mild** | **Moderate** | **Severe** | **Convalescent** |
|  | **(N = 20)** | **(N = 14)** | **(N = 12)** | **(N = 34)** | **(N = 18)** | **(N = 33)** |
| **CD200** (pg/mL) | 181.30 (0–521.60) | 396.94 (154.14–564.53) | 584.44 (486.29–1332.82) | 507.37 (166.78–964.06) | 332.06 (139.25–498.33) | 510.46 (218.78–834.47) |
| **FABP2** (pg/mL) | 3.93 (0–225.13) | 262.51 (137.29–415.94) | 460.78 (261.45–908.37) | 226.87 (108.61–398.39) | 139.60 (46.26–245.35) | 289.52 (144.35–555.17) |
| **sFRP-3**  (ng/mL) | 0.20 (0–5.47) | 8.45 (5.09–15.60) | 12.43 (4.70–20.89) | 13.25 (7.24–25.09) | 10.64 (8.26–25.18) | 14.38 (7.17–22.54) |
| **TRAIL R1**  (pg/mL) | 0 (0–640.29) | 606.01 (465.76–998.93) | 1100.13 (787.49–4965.73) | 620.71 (279.57–2528.37) | 486.02 (163.32–853.89) | 612.85 (260.05–1476.11) |
| **Transferrin**  (ng/mL) | 13.53 (0.70–25.20) | 36.23 (14.64–46.48) | 34.92 (25.88–54.69) | 36.08 (19.95–54.34) | 40.27 (15.61–48.68) | 48.65 (33.49–56.93) |
| **IL-1F7**  (pg/mL) | 339.01 (101.12–551.54) | 0 (0–137.70) | 24.98 (0–266.00) | 0 (0–299.89) | 0 (0–181.95) | 0 (0–216.12) |
| **IL-5Rα**  (pg/mL) | 958.73 (518.50–1343.91) | 0 (0–168.68) | 0 (0–453.37) | 0 (0–1131.89) | 0 (0–654.62) | 0 (0–218.65) |
| **GDF15**  (pg/mL) | 13.55 (8.05–79.07) | 151.25 (41.39–249.21) | 136.45 (44.70–321.41) | 256.24 (76.17–341.00) | 524.85 (405.14–831.19) | 207.46 (95.03–282.49) |
| **IGFBP-1**  (ng/mL) | 0.19 (0.12–1.63) | 3.13 (0.75–4.93) | 2.78 (0.61–6.22) | 1.26 (0.64–2.66) | 2.87 (1.40–5.00) | 2.81 (1.24–3.57) |
| **IGFBP-4**  (ng/mL) | 3.42 (0–23.01) | 116.99 (52.67–279.43) | 56.41 (17.78–155.00) | 81.21 (24.79–162.18) | 237.01 (140.92–459.39) | 123.29 (62.44–226.35) |
| **VCAM-1**  (ng/mL) | 13.63 (6.07–27.68) | 66.07 (39.32–90.15) | 55.15 (40.71–66.25) | 43.38 (36.03–68.56) | 58.58 (42.92–74.53) | 49.55 (31.68–71.10) |
